# Heterologous prime-boost vaccination with VLA2001 and an ORFV-based vector enhances spike- and nucleocapsid-specific immunity in mice

**DOI:** 10.3389/fimmu.2025.1675859

**Published:** 2025-09-18

**Authors:** Alena Reguzova, Verena Haug, Carina Metz, Melanie Müller, Madeleine Fandrich, Alex Dulovic, Ralf Amann

**Affiliations:** ^1^ Institute of Immunology, University Hospital Tübingen, Tübingen, Germany; ^2^ Institute for Tropical Medicine, Travel Medicine, and Human Parasitology, University Hospital Tübingen, Tübingen, Germany; ^3^ NMI Natural and Medical Sciences Institute at the University of Tübingen, Reutlingen, Germany

**Keywords:** Orf virus, viral vector, vaccines, heterologous prime-boost, SARS-CoV-2, VLA2001

## Abstract

Heterologous prime–boost vaccination has emerged as a promising approach to enhance immune responses by combining vaccines with complementary mechanisms of antigen delivery and immune activation. Here, we evaluated the immunogenicity of heterologous regimens combining the licensed inactivated SARS-CoV-2 vaccine (VLA2001) with the replication-deficient Orf virus-based vector vaccine (Prime-2-CoV). Using a mouse model, we compared these regimens to homologous vaccinations with each vaccine alone. Among the combinations tested, priming with VLA2001 followed by boosting with Prime-2-CoV induced the strongest spike-specific antibody responses, superior ACE2-binding inhibition against pre-Omicron variants, and robust Th1-biased immunity, with robust CD4^+^ and CD8^+^ T-cell responses. This sequence also enhanced nucleocapsid-specific immunity, underscoring the benefit of multiantigen targeting. These findings highlight the immunological synergy between inactivated whole-virus and ORFV vector vaccines and support the strategic use of Prime-2-CoV as a potent heterologous booster. The ORFV platform’s favorable safety profile and Th1-polarizing capacity make it a valuable candidate for future heterologous vaccine strategies beyond SARS-CoV-2.

## 1 Introduction

Effective vaccination strategies remain central to controlling future outbreaks caused by emerging and re-emerging viruses (1, 2). While homologous vaccine regimens have been widely deployed, they may be limited by antigenic imprinting and a narrow breadth of immune responses, which can reduce efficacy against viral variants (3). Furthermore, repeated administration of the same vaccine type may lead to diminished immune stimulation due to vector-specific immunity or immune exhaustion (4–6).

To address these challenges, heterologous prime-boost vaccination, the sequential administration of vaccines with different underlying technologies, has emerged as a promising alternative (7–10). During the COVID-19 pandemic, this strategy was extensively tested and has shown to enhance immunogenicity in both preclinical and clinical settings. For example, priming with an adenoviral vector vaccine followed by a boost with Pfizer-BioNTech’s mRNA vaccine BNT162b2 elicited significantly stronger immune responses than homologous regimens (9–13). Several studies reported that heterologous combinations induced up to 20- to 60-fold higher neutralizing antibody titers and more robust T-cell responses (9–11). Importantly, the benefits of heterologous vaccination extend beyond immunogenicity. By combining platforms with distinct mechanisms of action and delivery characteristics, such regimens can leverage complementary strengths—enhancing protective efficacy, balancing reactogenicity, improving safety profiles, and facilitating vaccine distribution through optimized storage or manufacturing requirements. Thus, heterologous regimens represent a promising evolution in vaccine strategy. However, empirical evidence supporting the use of heterologous regimens across a broader range of vaccine platforms remains limited (3).

Inactivated whole-virus vaccines remain an essential part of the global immunization toolkit. They offer broad immune activation, excellent safety profiles, even in vulnerable populations, and practical advantages in storage and distribution. However, because they rely on exogenously administered antigens, classical inactivated vaccines typically induce strong humoral responses but elicit only limited cellular immunity. To overcome this limitation, advanced formulations incorporate immunostimulatory adjuvants. A prominent example is Valneva’s inactivated SARS-CoV-2 vaccine VLA2001 (14). It is formulated with two adjuvants: aluminum hydroxide (Alum) and CpG 1018. Alum promotes antigen uptake and presentation and drives a Th2-skewed immune response, while CpG 1018, a Toll-like receptor 9 (TLR9) agonist, activates innate immune cells and promotes Th1-biased responses (15). Together, these adjuvants are intended to enhance immunogenicity and induce a more balanced humoral and cellular immune response.

**Figure 1:**
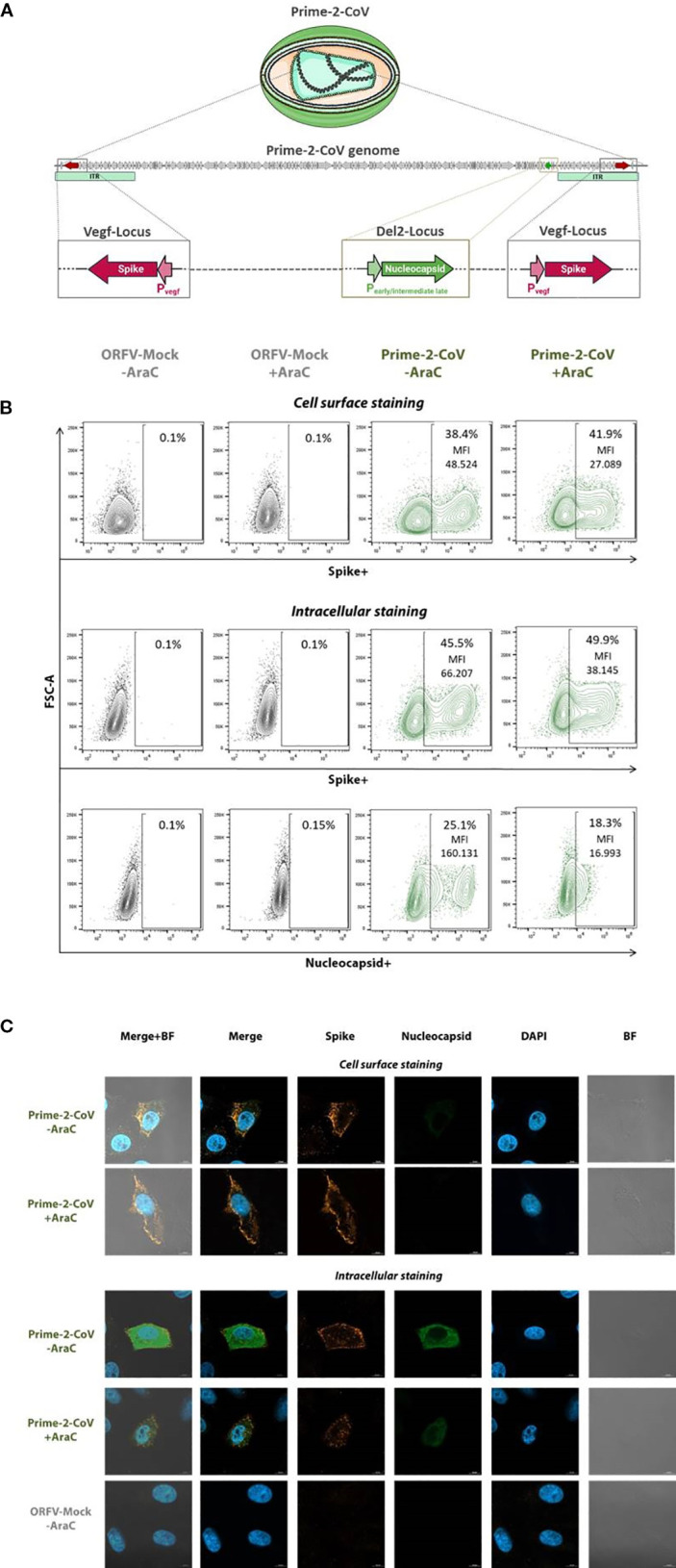
*In vitro* characterization of Prime-2-CoV antigen expression. **(A)** Schematic representation of Prime-2-CoV encoding the full-length SARS-CoV-2 spike and nucleocapsid proteins. **(B)** Vero cells were infected with Prime-2-CoV (MOI 1) for 16 h in the presence or absence of cytosine arabinoside (AraC) to inhibit viral replication. Mock-infected cells served as controls. Spike and nucleocapsid expression were analyzed by flow cytometry, both on the cell surface and intracellularly, using specific monoclonal antibodies. Data shown for viable cells with mean fluorescence intensity (MFI) values indicated for Prime-2-CoV–infected samples. **(C)** Fluorescence microscopy images depicting spike and nucleocapsid expression in infected cells.

We previously described a novel vaccine platform based on a highly attenuated Orf virus vector (ORFV), derived from the D1701-VrV strain of the *Parapoxvirus* genus (16, 17). Current evidence implicates roles for macropinocytosis and clathrin-mediated endocytosis in cell entry, with no single dedicated receptor has been established to date (18, 19). ORFV replicates exclusively in the cytoplasm, avoids integration into the host genome, and has a restricted host tropism. Importantly, in human and murine cells ORFV is non-permissive, restricting late gene expression and progeny formation, contributing to its excellent safety profile (20). In addition, it encodes multiple immunomodulatory genes that help shape innate and adaptive responses (21). The ORFV platform uniquely enables effective re-immunizations due to its characteristic of inducing only short-lived vector-specific immunity, while it can elicit strong and durable immune responses to vector-encoded antigens (21–29). Prime-2-CoV is a multi-antigenic ORFV-based vaccine candidate targeting the SARS-CoV-2 spike and nucleocapsid proteins; the vector´s architecture and promoter usage are illustrated in **Figure 1A**. It has demonstrated strong immunogenicity in mice, hamsters and non-human primates (16), shown no *in vivo* replication and rapid viral clearance in rats and mice (20), as well as an excellent safety and immunogenicity profile in first-in-human, phase I trials (17, 30).

To explore the potential immunological synergy between these two complementary vaccine technologies, we evaluated heterologous prime–boost regimens combining Prime-2-CoV and VLA2001 using SARS-CoV-2 as a clinically relevant and immunologically well-characterized model. We compared this heterologous strategy with homologous two-dose regimens in which each vaccine was administered alone. Humoral and cellular immune responses were assessed, including neutralization capacity against several SARS-CoV-2 variants, to determine whether the combination elicited an improved immunogenicity profile. Our findings support the concept that heterologous vaccination can enhance immune protection by leveraging the distinct strengths of different vaccine platforms.

## 2 Materials and methods

### 2.1 *In vitro* transgene expression

To evaluate antigen expression by the ORFV-based vaccine vector Prime-2-CoV via flow cytometry, Vero cells were seeded into 24-well plates (Greiner Bio-One, Frickenhausen, Germany) at a density of 2 × 10^5^ cells per well and infected at a multiplicity of infection (MOI) of 1 with either Prime-2-CoV or a mock control ORFV. Where indicated, cells were pre-treated with 40 µg/mL cytosine arabinoside (AraC; Sigma-Aldrich, St. Louis, MO, USA) for 30 minutes prior to infection. Sixteen hours post-infection, cells were harvested for analysis. Cell viability was assessed using Zombie Aqua Fixable Viability Dye (cat. no. 423102, BioLegend, San Diego, CA, USA). Surface staining was performed using a SARS-CoV-2 spike-specific rabbit monoclonal antibody (cat. no. 40592-R001, Sino Biological, Eschborn, Germany) in combination with an in-house generated AF647 anti-ORFV antibody. Cells were then fixed and permeabilized using Fixation & Permeabilization Solution (BD Biosciences, Franklin Lakes, NJ, USA), followed by intracellular staining with a SARS-CoV-2 nucleocapsid-specific mouse monoclonal antibody (cat. no. GTX135357, GeneTex, Irvine, CA, USA). Detection was performed using fluorophore-conjugated secondary antibodies: Alexa Fluor 555 anti-rabbit IgG (cat. no. A32732, Thermo Fisher Scientific, Waltham, MA, USA) and Alexa Fluor 488 anti-mouse IgG (cat. no. A11029, Thermo Fisher Scientific). Samples were acquired on a BD LSR Fortessa flow cytometer (BD Biosciences) and analyzed using FlowJo^®^ software v10 (BD Biosciences).

For immunofluorescence microscopy, 4 × 10^4^ Vero cells were seeded into an 8-well chamber slide (ibidi GmbH, Gräfelfing, Germany) and infected with either Prime-2-CoV or a mock control at an MOI of 1, with or without pre-treatment using 40 µg/mL AraC for 30 minutes. Sixteen hours post-infection, cells were fixed with 4% methanol-free formaldehyde (Thermo Fisher Scientific) for 15 minutes at room temperature, permeabilized with 0.2% Triton X-100 in PBS for 5 minutes, and blocked with 5% BSA in PBS. Cells were then incubated for 2 hours at room temperature with primary antibodies specific for SARS-CoV-2 spike (cat. no. 40592-R001, Sino Biological) and nucleocapsid (cat. no. GTX135357, GeneTex), followed by incubation with secondary antibodies: Alexa Fluor 555 anti-rabbit IgG (cat. no. A32732, Thermo Fisher Scientific) and Alexa Fluor 488 anti-mouse IgG (cat. no. A11029, Thermo Fisher Scientific). Nuclei were counterstained using NucBlue Live Cell Stain (Invitrogen, Thermo Fisher Scientific), and slides were mounted with ibidi Mounting Medium. Fluorescence images were acquired using a Zeiss LSM800 confocal microscope equipped with a 63× oil immersion objective and processed using ZEN Blue 3.0 software (Carl Zeiss Microscopy GmbH, Oberkochen, Germany).

### 2.2 Ethics and animals

Animal housing and experimental procedures were conducted in strict accordance to Federation of European Laboratory Animal Science Associations recommendations and followed the guidelines of the Regional councils. Experiments were conducted by Synovo GmbH, Tübingen, Germany and all animal procedures (including surgery, anesthesia and euthanasia, as applicable) used in the current study were approved by the regional authority under the Project License Nr. 35/9185.81-7/SYN 12/20.

### 2.3 Immunization

The ORFV-based vaccine vector Prime-2-CoV, encoding the SARS-CoV-2 spike and nucleocapsid proteins of the ancestral Wuhan strain, was prepared as previously described (16). The inactivated SARS-CoV-2 vaccine VLA2001 (Valneva SE, Saint-Herblain, France) was purchased and used according to the manufacturer’s instructions. Female CD-1 mice (Charles River Laboratories, Sulzfeld, Germany), aged 7–9 weeks, were randomly assigned to experimental groups (n = 5–7 per group).

We chose an outbred strain to better approximate genetic diversity and reduce strain-specific biases in humoral and T-cell readouts, which is common practice in vaccine immunogenicity screens (31). Mice were immunized intramuscularly (i.m.) on days 0 and 21 with either 1/10 of the human dose of VLA2001 or 1 × 10^6^ plaque-forming units (PFU) of Prime-2-CoV. The selected dose of Prime-2-CoV corresponds to approximately 1/100 of the human dose (1 × 10^8^ PFU). The intramuscular route was chosen to reflect clinical use of both platforms and to enable a head-to-head immunogenicity comparison under clinically relevant conditions. Peripheral blood was collected from the tail vein under isoflurane anesthesia (3–4% in O_2_) on days 14, 21, and 28. At the experimental endpoint (day 35), mice were anesthetized with isoflurane and euthanized via cardiac puncture. Blood was collected, and spleens were harvested for downstream analyses. Splenocytes were isolated and cryopreserved using standard procedures.

### 2.4 Detection of specific serum IgG by ELISA

Spike-, nucleocapsid (N)- and ORFV-binding antibodies were quantified in mouse serum samples using an indirect enzyme-linked immunosorbent assay (ELISA). Nunc Maxisorp 96-well plates (Fisher Scientific, Schwerte, Germany) were coated overnight at 4°C with 5 μg/ml of recombinant SARS-CoV-2 full-length spike protein (cat. no. 40589-V08B1, Sino Biological) or N protein (cat. no. 40588-V08B, Sino Biological) or 10^7^ PFU/ml of ORFV in PBS (Thermo Fisher Scientific). Plates were then blocked for 2 hours at room temperature with 3% bovine serum albumin (BSA; Carl Roth, Karlsruhe, Germany) in PBS to prevent non-specific binding. Afterwards, serial dilutions of serum samples were added to the wells and incubated for 1 hour at room temperature. Antigen-bound antibodies were detected using horseradish peroxidase (HRP)-conjugated goat anti-mouse antibodies specific for total IgG (1:5000, Abcam, Cambridge, UK, ab6728), IgG1 (1:1000, Abcam, ab97240), or IgG2a (1:1000, Abcam, ab97245). Following a 1-hour incubation, plates were developed using TMB substrate (Cat. No. 421101, BioLegend) and the reaction was stopped after sufficient color development by adding stop solution (cat. no. 423001, BioLegend). All samples and controls were run in duplicate. Absorbance was measured at 450 nm, and background values (blank wells) were subtracted from sample readings. Endpoint titers were determined by plotting the log_10_-transformed optical density (OD) values against the log_10_ of the serum dilution. A linear regression model was applied, and the endpoint titer was defined as the dilution at which the regression line of the sample intersected with the OD cut-off value of 0.1.

### 2.5 RBDCoV-ACE2

RBDCoV-ACE2, a previously published multiplex ACE2 inhibition assay (32), analyses neutralizing antibody activity using ACE2 binding inhibition as a surrogate. Neutralizing antibodies were analyzed against the SARS-CoV-2 WT, Beta, Delta, Omicron BA2 and XBB.1.5 variants. Samples were measured at dilution factor of 1:400. In brief, RBD variant proteins (33–36) were coupled to spectrally distinct populations of MagPlex beads (cat. no. MC100XX, Luminex, Austin, TX, USA) and then combined into a bead mix. Serum samples were diluted with assay buffer and then ACE2 buffer (300 ng/mL biotinylated ACE2, cat. no. 10108-H08H-B, Sino Biological), before being combined 1:1 with the bead mix in 96 well plates (cat. no. 3642, Corning, Corning, NY, USA). After incubation for 2 hours at 21°C, 750rpm in a thermomixer, the beads were washed 3x in wash buffer using an automated microplate washer (Biotek 405TS, Winooski, VT, USA). Bound ACE2 was detected using 2ug/mL Strep-PE (cat. no. SAPE-001, Moss, Pasadena, MD, USA) by incubating the bead-sample mix for a further 45 mins. Following a further washing step, the beads were resuspended in 100uL of wash buffer, shaken for 3 mins at 1000rpm and then measured on a FLEXMAP3D using the following settings: Timeout 80 sec, Gate 7500-15000, Reporter Gain: Standard PMT, 50 events. As controls, 150ng/mL ACE2, blanks and 2 QC samples (all in duplicate) were included. ACE2 binding inhibition (%) was calculated as a percentage, with 100% indicating maximum ACE2 binding inhibition and 0% no ACE2 binding inhibition.

### 2.6 Intracellular cytokine staining

Cryopreserved splenocytes were thawed, rested for 4 hours at 37°C in complete RPMI medium, and seeded into 96-well round-bottom plates (Greiner Bio-One, Frickenhausen, Germany) at a density of 2 × 10^6^ cells per well. Cells were re-stimulated with 0.5 μg/mL of SARS-CoV-2 full-length spike (PM-WCPV-S-1) or nucleocapsid (PM-WCPV-NCAP-1) peptide pools (both JPT Peptide Technologies, Berlin, Germany) in the presence of 1 μg/mL anti-mouse CD28 (cat. no. 102116) and CD49d (cat. no. 103710) co-stimulatory antibodies (both BioLegend). After 1 hour of stimulation, Brefeldin A (10 μg/mL; Sigma-Aldrich, St. Louis, MO, USA), Monensin (cat. no. 420701, BioLegend), and anti-mouse CD107a antibody (cat. no. 121620, BioLegend) were added, and cells were incubated for an additional 14 hours at 37°C. Following stimulation, cells were blocked with TruStain FcX™ (anti-mouse CD16/32, cat. no. 101320, BioLegend) for 10 minutes at room temperature and subsequently stained for 30 minutes at room temperature with a surface antibody cocktail containing anti-CD3ϵ (cat. no. 100312), CD4 (cat. no. 100531), CD8α (cat. no. 100730), CD62L (cat. no. 104430), and CD44 (cat. no. 103022), together with Zombie Aqua™ Fixable Viability Dye (cat. no. 423102, all BioLegend). Cells were then fixed and permeabilized using Fixation & Permeabilization Solution (BD Biosciences) for 30 minutes and stained intracellularly for cytokines with anti-mouse TNF-α (cat. no. 506344), IFN-γ (cat. no. 505835), IL-2 (cat. no. 503808), IL-4 (cat. no. 504104), and IL-17A (cat. no. 506941) antibodies (all BioLegend) for 30 minutes at 4°C. Samples were acquired on an Attune NxT flow cytometer (Thermo Fisher Scientific) and analyzed using FlowJo^®^ v10 software (BD Biosciences). Background responses from unstimulated control wells were subtracted from peptide-stimulated conditions.

### 2.7 Germinal center B cells and T follicular helper cells

Cryopreserved splenocytes were thawed and rested for 4 hours at 37°C in complete RPMI medium. To block Fc receptors, cells were incubated with TruStain FcX™ (anti-mouse CD16/32, cat. no. 101320, BioLegend) for 10 minutes at room temperature. Surface staining was then performed for 30 minutes at 4°C using an antibody cocktail targeting the following markers: CD3ϵ (cat. no. 100320), CD4 (cat. no. 100531), CD8α (cat. no. 100730), CD62L (cat. no. 104406), CD44 (cat. no. 103032), CD19 (cat. no. 115530), CXCR5 (cat. no. 145529), PD-1 (cat. no. 135220), and GL7 (cat. no. 144608), along with Zombie Aqua™ Fixable Viability Dye (cat. no. 423102, all BioLegend). Samples were acquired on an Attune NxT flow cytometer (Thermo Fisher Scientific) and analyzed using FlowJo^®^ v10 software (BD Biosciences).

### 2.8 Statistical analysis

Statistical analyses were performed using GraphPad Prism version 9 (GraphPad Software, San Diego, CA, USA). Normality of data distributions was assessed using the Shapiro–Wilk test. Group comparisons were conducted using the Mann–Whitney U test or Kruskal–Wallis test, as appropriate. P-values < 0.05 were considered statistically significant. Statistical significance is indicated as follows: *p < 0.05; **p < 0.01; ***p < 0.001; ****p < 0.0001. Data are presented as means ± standard error of the mean (SEM), geometric mean with geometric standard deviation (SD), or medians, as indicated in the figure legends.

## 3 Results

### 3.1 Spike and nucleocapsid antigen expression from Prime-2-CoV

The Prime-2-CoV vaccine candidate used in this study was previously described (16) and encodes the full-length spike (S) protein of ancestral SARS-CoV-2 (Wuhan strain) (37) under the control of the early ORFV Pvegf promoter. The spike sequence incorporates key stabilizing modifications: the D614G substitution, the K986P/V987P double proline mutation, and a deletion of the furin cleavage site (RRAR, residues 682–685), replaced by GSAS to stabilize the protein in its prefusion conformation (38). In addition, the nucleocapsid (N) gene is expressed under the control of an artificial early/moderate late P7 promoter.

To confirm antigen expression, Vero cells were infected with Prime-2-CoV at a multiplicity of infection (MOI) of 1 for 16 hours in the presence or absence of cytosine arabinoside (AraC), a DNA synthesis inhibitor that blocks late viral gene expression. ORFV-Mock-infected cells served as controls.

Flow cytometry analysis revealed robust spike antigen production, detectable both at the cell surface and intracellularly (**Figure 1B**). The presence of AraC moderately reduced spike-specific mean fluorescence intensity (MFI) by approximately 1.8-fold, suggesting a minor contribution from late-phase promoter activity. However, the high residual MFI observed in the presence of AraC strongly indicates predominant expression driven by early-phase promoters. In contrast, N antigen was exclusively detected intracellularly, and AraC treatment markedly reduced N-specific MFI by approximately 9.5-fold, consistent with expression primarily driven by the early/moderate-late P7 promoter.

These findings were supported by fluorescence microscopy, demonstrating spike localization on the cell surface and intracellular expression of both spike and N proteins, consistent with observations from flow cytometry (**Figure 1C**).

### 3.2 Spike-specific IgG responses

**Figure 2:**
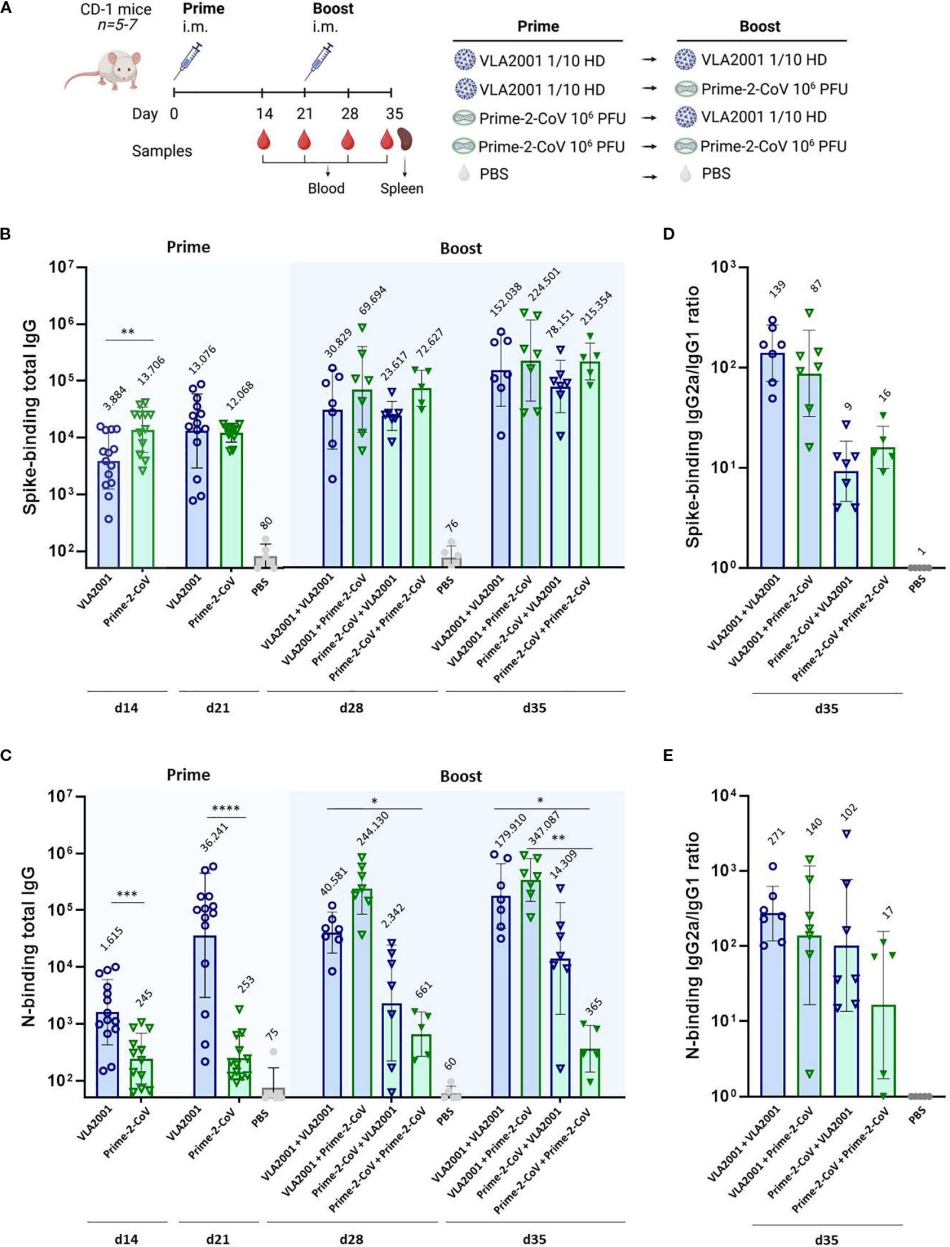
Humoral immune responses following homologous and heterologous immunization with Prime-2-CoV and VLA2001 in CD-1 mice. **(A)** Schematic overview of the experimental design, including group allocation, vaccination schedule, and sample collection time points. **(B)** Endpoint titers of spike-specific total IgG in mouse serum measured by ELISA at 2 and 3 weeks after the first immunization (days 14 and 21) and 1 and 2 weeks after the second immunization (days 28 and 35). **(C)** Endpoint titers of Nucleocapsid (N)-specific total IgG in serum assessed at the same time points. **(D)** Ratio of IgG2a to IgG1 isotypes among spike-specific antibodies on day 35, indicating Th1/Th2 polarization. **(E)** IgG2a/IgG1 ratio of N-specific antibodies on day 35. In **(B–F)** data are presented as geometric mean values ± geometric standard deviation (SD). Geometric mean titers (GMT) are noted above the columns. *p < 0.05; **p < 0.01; ***p < 0.001; ****p < 0.0001.

To assess the immunogenicity of heterologous prime-boost vaccination regimens combining a novel Orf virus-based SARS-CoV-2 vaccine Prime-2-CoV with the inactivated and adjuvanted whole-virus SARS-CoV-2 vaccine Valneva VLA2001, we immunized mice using various homologous and heterologous prime-boost combinations (**Figure 2A**).

We first evaluated spike-specific IgG responses by ELISA. A single immunization with 10^6^ PFU of Prime-2-CoV—approximately 1/100 of the intended human dose—induced significantly higher spike-specific IgG levels two weeks post-immunization compared to a 1/10 human dose of VLA2001, highlighting the strong immunogenicity of the Prime-2-CoV vaccine and the ORFV vector platform (**Figure 2B**). Notably, boosting Prime-2-CoV-primed animals with VLA2001 did not enhance antibody levels, indicating a lack of additive or synergistic effect in this direction of the combination. In contrast, the reverse sequence—priming with VLA2001 followed by a Prime-2-CoV boost—resulted in a two-fold increase in spike-specific IgG compared to the homologous VLA2001 regimen. This suggests a benefit of using Prime-2-CoV as a booster following whole-virus priming. Remarkably, the heterologous VLA2001/Prime-2-CoV regimen elicited antibody levels comparable to those induced by two doses of Prime-2-CoV, further emphasizing the potent humoral response elicited by Prime-2-CoV in both homologous and heterologous settings.

### 3.3 Nucleocapsid-specific IgG responses

We next examined antibody responses against the N protein. Here, we observed a distinct pattern. As a whole-virus vaccine, VLA2001 effectively primed N-specific IgG responses, with significantly higher levels than a single dose of Prime-2-CoV (**Figure 2C**). However, boosting with Prime-2-CoV led to a marked enhancement of these responses. The heterologous VLA2001/Prime-2-CoV regimen induced significantly higher N-specific IgG levels than either of the homologous regimens. This effect was not observed in the homologous Prime-2-CoV group, which induced relatively moderate N-specific IgG responses, indicating that the sequential exposure to inactivated and vector-delivered antigen has a boosting effect on the N-specific humoral arm.

### 3.4 IgG subclass profiles and Th1 bias

To evaluate the qualitative nature of the humoral response and infer T helper cell polarization, we assessed the ratio of IgG2a to IgG1 in serum samples. All vaccination regimens elicited a predominant IgG2a response over IgG1, indicative of a Th1-skewed immune profile (**Figures 2D, E**). This Th1 bias was observed for antibodies directed against both spike and N antigens and is consistent with an antiviral immune response known to support viral clearance and cytotoxic T cell activation. Interestingly, the Th1 polarization was particularly pronounced in groups receiving the inactivated vaccine VLA2001, either alone or in heterologous combination, which is likely attributable to the CpG1018 adjuvant in Valneva´s formulation.

### 3.5 ORFV-specific IgG responses

To assess the immunogenicity of the ORFV vector itself, we measured antibody responses against Prime-2-CoV. A single immunization induced detectable ORFV-specific IgG in mice, with markedly increased titers observed following a second homologous dose (**Supplementary Figure S1A**). Importantly, the presence of anti-ORFV antibodies did not impair the boosting and kinetic of spike- or N-specific IgG responses in homologous regimen (**Supplementary Figures S1B, C**), suggesting that pre-existing anti-vector immunity does not limit the immunogenicity of subsequent antigen delivery by Prime-2-CoV.

### 3.6 ACE2 binding inhibition of SARS-CoV-2 variants of concern

**Figure 3:**
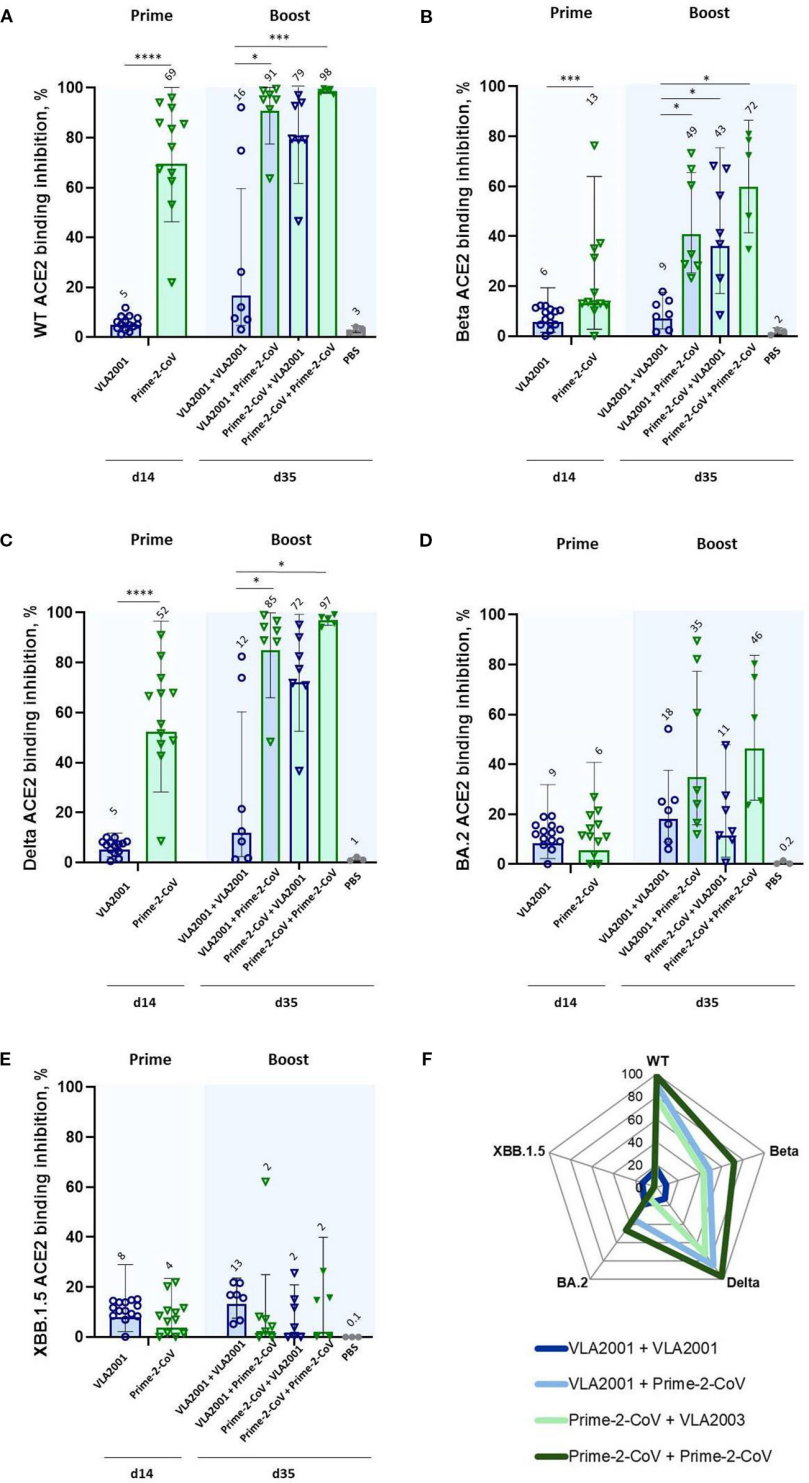
ACE2 binding inhibition against SARS-CoV-2 variants of concern (VoC) following homologous and heterologous immunization with Prime-2-CoV and VLA2001 in CD-1 mice. ACE2 binding inhibition (%) in serum was measured at two time points: day 14 (two weeks after the first immunization) and day 35 (two weeks after the second immunization). Inhibition was assessed against the following RBD variants: **(A)** Wild-type (WT), **(B)** Beta, **(C)** Delta, **(D)** Omicron BA.2, and **(E)** Omicron XBB.1.5. Data are presented as geometric mean values ± geometric SD. GMT are indicated above the bars. **(F)** Radar chart displaying geometric mean ACE2 binding inhibition (%) in serum on day 35 across all five variants. All samples were tested at a fixed serum dilution of 1:400. Responses <0.1% were set to 0.1% for visualization purposes; values >20% are considered indicative of a positive response. *p < 0.05; ***p < 0.001; ****p < 0.0001.

We next evaluated the functional capacity of vaccine-induced antibodies to inhibit ACE2 binding to the receptor-binding domain (RBD) of SARS-CoV-2 VoC using the RBDCoV-ACE2 assay. Two weeks after a single immunization, serum from animals receiving 10^6^ PFU of Prime-2-CoV showed significantly higher levels of ACE2 binding inhibition against “historical,” pre-Omicron VoC compared to animals receiving a 1/10 human dose of VLA2001 (**Figures 3A–C**), mirroring the trends observed for spike-specific IgG responses and underscoring the strong immunogenicity of Prime-2-CoV. As anticipated, ACE2 binding inhibition against more recent Omicron-lineage VoC was substantially lower across all groups after a single immunization (**Figures 3D, E**).

Homologous boosting with VLA2001 did not appreciably increase ACE2 binding inhibition relative to priming alone. In contrast, heterologous boosting of VLA2001-primed animals with Prime-2-CoV significantly enhanced ACE2 binding inhibition of pre-Omicron VoC, indicating a synergistic effect of this prime-boost direction. While boosting Prime-2-CoV-primed animals with VLA2001 also improved responses, the effect was less pronounced, reinforcing the superior boosting capacity of Prime-2-CoV.

Among all tested regimens, two doses of Prime-2-CoV induced the highest levels of ACE2 binding inhibition against 4 out of 5 tested VoC (**Figure 3F**), further supporting the broad neutralization capacity and strong humoral immunogenicity of the Prime-2-CoV vaccine.

### 3.7 Germinal center B cells and Tfh cell responses

The formation of germinal centers (GCs) and interactions with T follicular helper (Tfh) cells are crucial for the generation of high-affinity antibodies and long-lived memory B cells. We evaluated the presence of GC B cells and their interaction with Tfh cells in spleen. All vaccine regimens, regardless of combination, induced comparable frequencies of GC B cells and Tfh cells engaging in GC interactions (**Supplementary Figure S2A**). This indicates that while overall antibody levels differed between regimens, the capacity to establish the necessary cellular infrastructure for sustained humoral memory was not significantly affected by vaccine type or sequence.

### 3.8 CD4^+^ T cell responses

**Figure 4:**
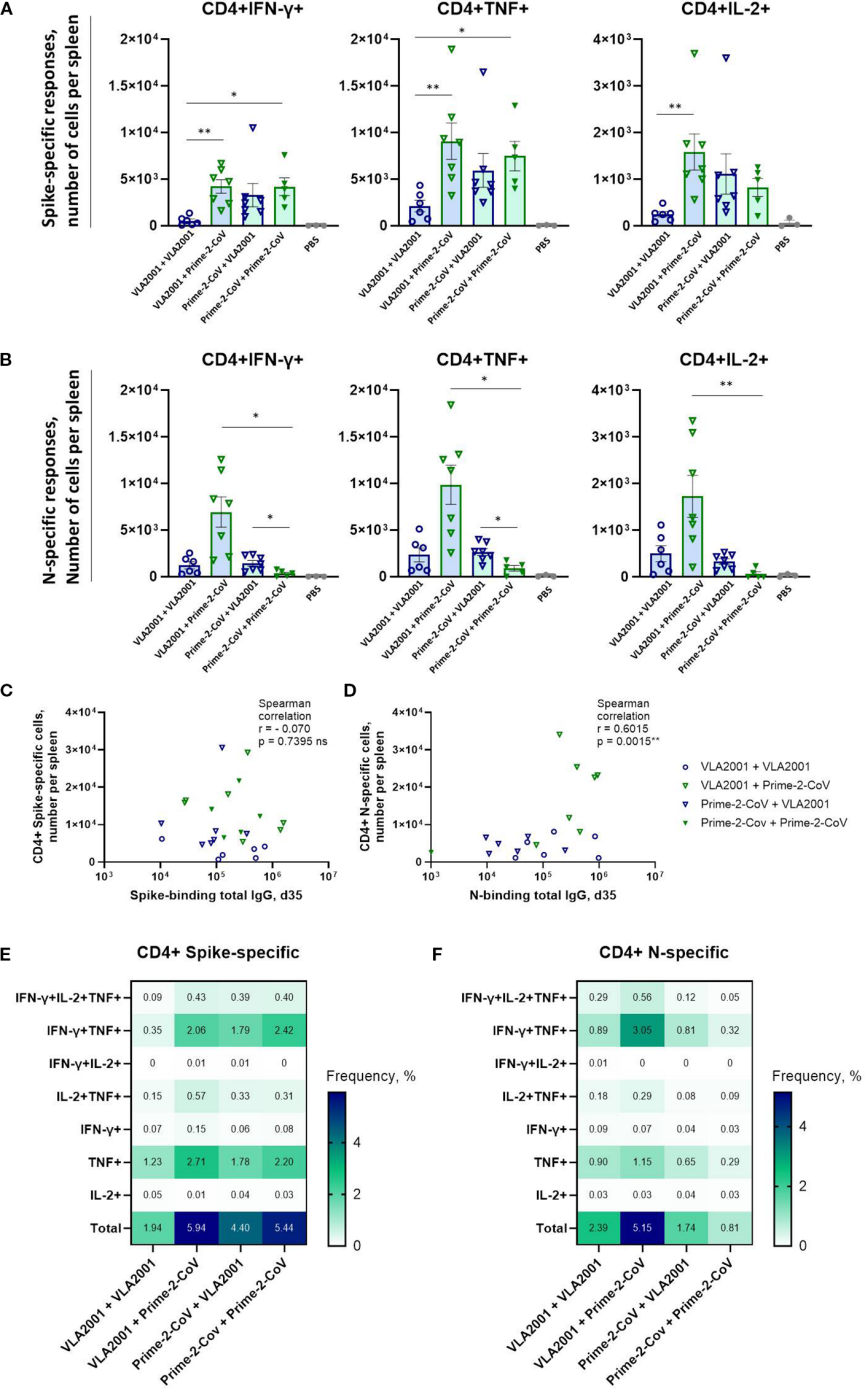
Antigen-specific CD4^+^ T cell immune responses induced by homologous and heterologous vaccination with Prime-2-CoV and VLA2001 in CD-1 mice. CD-1 mice were immunized on days 0 and 21 with 10^6^ PFU of Prime-2-CoV, 1/10 of the human dose of VLA2001, or PBS as control. **(A, B)** Numbers of CD4^+^ T cells producing cytokines per spleen in response to *ex vivo* stimulation with SARS-CoV-2 **(A)** Spike or **(B)** nucleocapsid (N) peptide pools, measured in splenocytes on day 35 by intracellular cytokine staining (ICS). **(C, D)** Correlation between the numbers of antigen-specific CD4^+^ T cells per spleen and the corresponding total IgG endpoint titers in serum on day 35: **(C)** spike-specific responses and **(D)** N-specific responses. In **(A, B)** heights of bars indicate mean ± SEM (standard error of the mean). **(E, F)** Heatmaps depict the mean frequencies of polyfunctional **(E)** spike-specific CD4^+^ T cells and **(F)** N-specific CD4^+^ T cells per group, measured in splenocytes on day 35. *p < 0.05; **p < 0.01.

To further characterize the cellular arm of the immune response, we analyzed antigen-specific CD4^+^ T cells by intracellular cytokine staining (ICS) following restimulation with spike or N peptide pools. Spike-specific CD4^+^ T cell responses were consistently low in the homologous VLA2001 group, whereas regimens containing the Prime-2-CoV vaccine induced markedly higher frequencies of cytokine-producing CD4^+^ T cells (**Figure 4A**). Both the heterologous VLA2001/Prime-2-CoV and homologous Prime-2-CoV regimens elicited robust spike-reactive CD4^+^ responses. However, the heterologous combination did not exceed the response induced by two doses of Prime-2-CoV, suggesting that CD4^+^ T cell immunogenicity is primarily driven by the ORFV vector.

In contrast, N-specific CD4^+^ T cell responses were highest in the heterologous VLA2001/Prime-2-CoV group, significantly exceeding those observed in either homologous regimen (**Figure 4B**). Notably, the homologous Prime-2-CoV group elicited only low levels of N-specific CD4^+^ T cells, while VLA2001 alone induced moderate responses, indicating that the inactivated whole-virus vaccine contributes to N-specific T cell priming. The enhanced response in the heterologous group suggests that a VLA2001 prime effectively initiates N-specific immunity, which is further boosted by Prime-2-CoV.

No correlation was observed between spike-specific CD4^+^ T cell frequencies and spike-specific IgG levels on day 35 (**Figure 4C**), suggesting distinct regulatory mechanisms or kinetics for cellular and humoral responses to spike. Conversely, a positive correlation was observed between N-specific CD4^+^ T cell frequencies and N-specific IgG levels (**Figure 4D**), indicating coordinated induction of humoral and cellular immunity against nucleocapsid. Cytokine profiling of antigen-specific CD4^+^ T cells revealed predominant expression of IFN-γ, TNF, and IL-2, with minimal IL-4 or IL-17A production (**Figures 4A, B**; **Supplementary Figures S2B, C**), consistent with a Th1-polarized response, as further supported by elevated IgG2a/IgG1 subclass ratios.

Polyfunctionality analysis of the Th1-polarized response revealed that among spike-specific CD4^+^ T cells, the majority of cytokine-producing cells expressed either TNF alone or a combination of IFN-γ and TNF (**Figure 4E**). The homologous VLA2001 regimen induced the lowest frequency of double-positive cells, while heterologous boosting with Prime-2-CoV improved this response. The highest frequencies of IFN-γ^+^TNF^+^ CD4^+^ T cells were observed in the homologous Prime-2-CoV group. A similar pattern was seen for N-specific CD4^+^ T cells, with the highest polyfunctional response in the VLA2001/Prime-2-CoV group and the lowest in the homologous Prime-2-CoV group (**Figure 4F**).

### 3.9 CD8^+^ T Cell responses

CD8^+^ T cells play a central role in the elimination of virus-infected cells, particularly in respiratory infections. Spike- and N-specific CD8^+^ T cell responses were therefore assessed by ICS following peptide restimulation.

**Figure 5:**
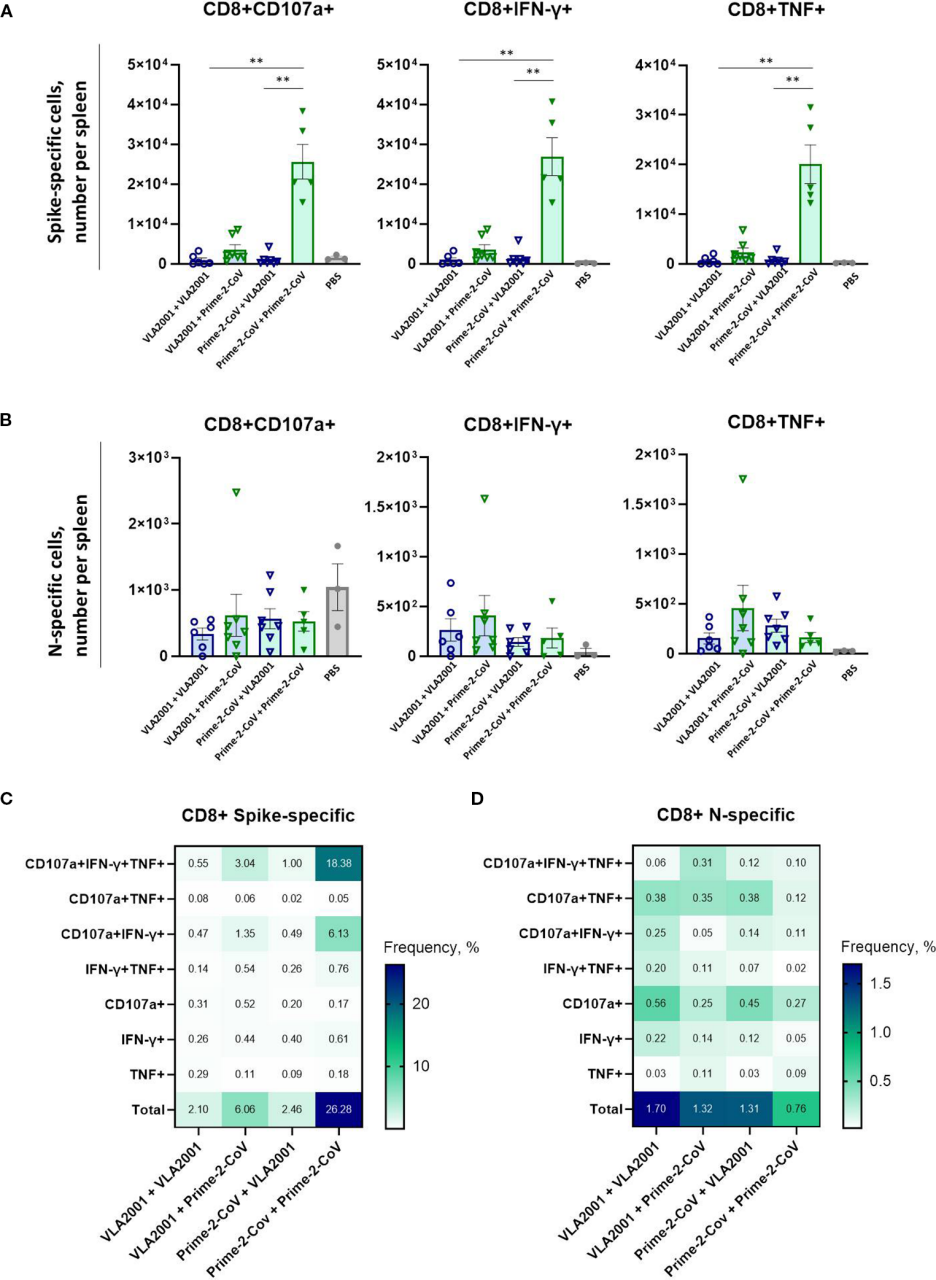
Antigen-specific CD8^+^ T cell immune responses induced by homologous and heterologous vaccination with Prime-2-CoV and VLA2001 in CD-1 mice. CD-1 mice were immunized on days 0 and 21 with 10^6^ PFU of Prime-2-CoV, 1/10 of the human dose of VLA2001, or PBS as control. **(A, B)** Numbers of CD8^+^ T cells producing cytokines per spleen in response to *ex vivo* stimulation with SARS-CoV-2 A) Spike or B) nucleocapsid (N) peptide pools, measured in splenocytes on day 35 by intracellular cytokine staining (ICS). In **(A, B)** heights of bars indicate mean ± SEM (standard error of the mean). **(C, D)** Heatmaps depict the mean frequencies of polyfunctional **(C)** spike-specific CD8^+^ T cells and **(D)** N-specific CD8^+^ T cells per group, measured in splenocytes on day 35. **p < 0.01.

The homologous Prime-2-CoV regimen induced by far the strongest spike-specific CD8^+^ T cell response, with significantly higher frequencies than all other groups (**Figure 5A**). The heterologous VLA2001/Prime-2-CoV group also elicited robust responses, albeit lower than those seen with the homologous Prime-2-CoV regimen, highlighting the strong capacity of the ORFV vector to drive cytotoxic responses even when administered as a booster. All other groups generated only weak to moderate spike-specific CD8^+^ T cell responses.

In contrast, nucleocapsid-specific CD8^+^ T cell responses were generally low across all vaccination groups (**Figure 5B**), with no significant differences detected between regimens. The homologous Prime-2-CoV group showed the lowest levels of N-specific CD8^+^ T cell activation.

Polyfunctionality profiling revealed a high proportion of spike-specific CD8^+^ T cells co-expressing CD107a, IFN-γ, and TNF (**Figure 5C**). The homologous VLA2001 regimen again showed the weakest response, while heterologous boosting with Prime-2-CoV enhanced the proportion of triple-positive CD8^+^ T cells. The highest levels were observed in the homologous Prime-2-CoV group.

For N-specific CD8^+^ T cells, the highest total response was seen in the homologous VLA2001 group; however, this was primarily limited to CD107a^+^ cells (**Figure 5D**). A more diverse polyfunctional profile—particularly CD107a^+^IFN-γ^+^TNF^+^ cells—was most pronounced in the VLA2001/Prime-2-CoV group, suggesting that this combination most effectively supports multifunctional cytotoxic T cell responses against nucleocapsid.

## 4 Discussion

This study provides proof-of-concept that heterologous prime-boost vaccination combining the licensed inactivated SARS-CoV-2 vaccine VLA2001 with the novel ORFV-based vector vaccine Prime-2-CoV can enhance both humoral and cellular immune responses. We pre-specified an intramuscular, clinically aligned immunogenicity screen to test for sequence-dependent synergy between two distinct platforms. Among the combinations tested, priming with VLA2001 followed by boosting with Prime-2-CoV elicited the most robust and broadly reactive immune responses, with a favorable Th1 bias. These results underscore the versatility and immunopotency of ORFV vectors and support their use as adaptable components in future heterologous vaccination strategies.

A key observation is the strong spike-specific antibody response induced by the VLA2001/Prime-2-CoV combination. This heterologous regimen matched or exceeded responses observed with homologous Prime-2-CoV vaccination and outperformed homologous VLA2001. Notably, even a single low-dose immunization with Prime-2-CoV elicited stronger responses than a subdose of VLA2001, underscoring the potent immunogenicity of the ORFV-based vector. Importantly, Prime-2-CoV was particularly effective as a booster following VLA2001 priming, whereas the reverse sequence was less immunogenic. This order dependence likely reflects non-redundant platform biology—including distinct antigen production kinetics, compartmentalization of antigen presentation, and adjuvant signatures—that are optimally combined when whole-virus priming precedes vector boosting.

Consistent with these findings, ACE2 binding inhibition assays indicated superior neutralizing activity against pre-Omicron VoC in animals receiving Prime-2-CoV-containing regimens. The heterologous VLA2001/Prime-2-CoV combination outperformed homologous VLA2001, supporting a sequence-dependent improvement with this prime-boost strategy. Although neutralizing responses against Omicron-lineage VoC were generally reduced, homologous Prime-2-CoV vaccination achieved the highest inhibition among the groups, underscoring its broad neutralizing potential.

These differences are likely attributable to distinct antigen designs. Prime-2-CoV expresses a pre-fusion stabilized spike protein (D614G, K986P/V987P, furin site deletion) (38), which promotes surface display and heightened immunogenicity (16). In contrast, VLA2001 presents the native Spike protein within inactivated virions, where partial transition to the post-fusion conformation may occur. This conformational shift potentially masking critical epitopes and thereby diminishing the magnitude of spike-specific immune responses (14).

All Prime-2-CoV–containing regimens induced strong, polyfunctional CD4^+^ and CD8^+^ T-cell responses against spike, underscoring the vector’s inherent immunostimulatory properties and its ability to elicit a type I immune response, characterized by Th1-skewed helper and cytotoxic T-cell activity. Such responses are considered critical for effective viral clearance and long-term protection (39, 40).

In addition to spike, Prime-2-CoV also encodes the nucleocapsid antigen. While VLA2001 alone was able to prime nucleocapsid-specific IgG and CD4^+^ T cell responses, the heterologous VLA2001/Prime-2-CoV regimen significantly enhanced these responses, exceeding levels observed in both homologous groups. This suggests a synergistic effect of whole-virus priming and vector-mediated boosting. A dual-antigen targeting strategy may be particularly valuable in the context of ongoing viral evolution, where mutations in the spike protein can compromise the efficacy of vaccines targeting spike alone (2, 41). However, unlike spike-specific antibodies, which give the most accurate correlates of protection against infection by the SARS-CoV-2 (42), the role of N-specific antibodies remains less defined (43–45). Emerging evidence suggests that non-neutralizing antibodies can contribute to antiviral defense via Fc-mediated effector mechanisms. Dangi et al. showed that N-specific antibodies mediated NK cell–dependent cytotoxicity against infected cells (44), while Herman et al. linked N-specific features such as antibody-dependent complement deposition and FcγR2B binding to clinical benefits of convalescent plasma therapy (45). In contrast, Fahoum et al. reported that anti-N IgG triggered complement deposition on uninfected lung cells in COVID-19 patients, leading to bystander cell damage (46). Nakayama et al. demonstrated that anti-N IgG1 enhanced IL-6 production in myeloid cells via Fc receptor–dependent mechanisms, indicating antibody-dependent enhancement of cytokine responses (47). In our study, nucleocapsid was included as a second antigen to test whether platform combinations could broaden immunity. Functional characterization of N-specific antibodies, however, was beyond the scope of this pre-specified immunogenicity analysis, which focused on sequence-dependent synergy between two clinically relevant vaccine platforms.

In contrast to CD4^+^ T cells, CD8^+^ T cell responses to nucleocapsid were uniformly low across all groups. This likely reflects intrinsic antigen properties and suboptimal cross-presentation. In Prime-2-CoV, the nucleocapsid antigen is expressed under the control of a weak early/moderate-late promoter. *In vitro* experiments showed reduced expression following AraC treatment in Vero cells, consistent with a dominant late-phase expression pattern. Since ORFV late genes are not expressed in non-permissive antigen-presenting cells *in vivo*, this likely limits MHC-I presentation and impairs efficient CD8^+^ T cell priming. In contrast, the spike antigen, expressed from a strong early promoter and optimized for surface display, induced strong CD8^+^ responses, particularly in the homologous Prime-2-CoV group. Robust N-specific CD4^+^ responses are consistent with predominantly exogenous routing of nucleocapsid in our regimens. Whole-virus priming supplies abundant virion-associated N for endosomal processing and MHC-II loading, and antigen released during abortive ORFV infection is likewise taken up exogenously by professional APCs (48). By contrast, late-promoter–driven N expression in non-permissive cells yields little endogenous antigen for proteasome/TAP-dependent MHC-I presentation. Thus, N-specific CD8^+^ priming would rely on cross-presentation, which may occur but is generally limited without high antigen load (49).

These findings suggest that re-engineering nucleocapsid expression for stronger early-phase activity may enhance antigen availability and increase the magnitude of N-specific immune responses. However, elevated N expression has been associated with immunopathology in preclinical models and should be carefully evaluated (46, 50).

Consistent with earlier data (16), pre-existing ORFV immunity had no detrimental effect on spike- or nucleocapsid-specific IgG responses, supporting the use of Prime-2-CoV for repeated immunizations. Anti-ORFV antibody responses are typically short-lived and often non-neutralizing, enabling effective boosting with ORFV vectors (51). Natural reinfections with wild-type ORFV are well documented in livestock, consistent with the absence of durable sterilizing immunity (52, 53). In line with this, a recent first-in-human phase I trial of Prime-2-CoV_Beta detected no ORFV-neutralizing antibodies despite robust transgene-specific immunity, further supporting the feasibility of repeated administration (17).

All tested regimens induced a Th1-biased immune profile, as indicated by elevated IgG2a/IgG1 ratios and antigen-specific IFN-γ, TNF, and IL-2 secretion by CD4^+^ T cells. In schedules containing VLA2001, this polarization was likely further reinforced by the CpG 1018 adjuvant used in the licensed formulation (14).

Despite comparable frequencies of germinal-center B cells and T-follicular-helper cells in all groups, the heterologous VLA2001/Prime-2-CoV combination elicited the strongest antibody responses. Thus, while the key cellular machinery of affinity maturation is engaged by every regimen, the order and nature of the prime and boost decisively shape the magnitude and breadth of the humoral response. Pre-existing immunity to spike or nucleocapsid antigens does not influence the immunogenicity of either when delivered by whole-virus and ORFV vector platforms. We did not observe correlations between pre-boost N-specific IgG (day 21) and post-boost spike-specific IgG titers (day 35), or vice versa, nor between spike- and N-specific IgG titers after boost (**Supplementary Figures S3A-C**). This likely reflects antigen-specific response kinetics and platform-dependent presentation: spike and N are recognized by largely distinct B-cell compartments and were delivered in different immunological contexts. Spike-specific CD4^+^ T cell frequencies did not correlate with spike-specific IgG levels on day 35, potentially suggesting distinct regulatory mechanisms or kinetics for cellular and humoral responses to spike (**Figure 4C**). Conversely, a strong positive correlation was observed between N-specific CD4^+^ T cell frequencies and N-specific IgG levels, indicating antigen-specific helper milieu that benefits N-responses (**Figure 4D**). Heterologous prime-boost did not create a generalized cross-antigen “global help” in our study, as we observed no correlation between N-specific CD4^+^ T-cell numbers and spike-specific IgG titers or ACE2 binding inhibition, nor between spike-specific CD4^+^ T cells and N-specific IgG, suggesting that N- and spike-specific responses develop largely independently (**Supplementary Figures S3D-F**). Beneficial effect of CD4^+^ T helper cells on CD8^+^ T cell responses against N did not occur, likely due to MHC-II–biased priming of CD4^+^ T cells versus limited CD8^+^ cross-presentation (**Supplementary Figure S3G**). However, spike-specific CD4^+^ and CD8^+^ T cells showed a moderate positive correlation, consistent with helper CD4^+^ T-cell conditioning promoting stronger CD8^+^ responses (**Supplementary Figure S3H**). Together, these findings suggest largely independent antigen-specific immunity shaped by prime-boost design and platform context.

Our findings align with growing evidence supporting the immunological benefits of heterologous vaccination. For instance, Barros-Martins et al. (11) and Schmidt et al. (12) demonstrated that adenoviral priming followed by mRNA boosting elicited stronger humoral and cellular responses than homologous adenoviral regimens. Likewise, Liu et al. (54) showed that protein-based priming followed by an adenoviral boost enhanced cross-neutralizing antibodies and T cell responses in mice. Li et al. (55) further reported that combining an adenovirus vector (AdC68) with mRNA vaccines induced broad immunity against multiple SARS-CoV-2 variants, including Omicron.

Real-world and clinical studies have shown that inactivated vaccines can substantially benefit from heterologous boosting. For instance, priming with inactivated CoronaVac followed by a ChAdOx1-S booster induced stronger antibody responses compared to homologous CoronaVac vaccination, whereas the reverse sequence, ChAdOx1-S priming followed by CoronaVac boosting, was considerably less effective (56). Similarly, significantly improved immunogenicity has been observed when primary vaccination with the inactivated Sinopharm vaccine (BBIBP-CorV) was heterologously boosted with mRNA vaccines (57). However, enhanced responses are not guaranteed: in the phase-3 COV-COMPARE extension trial, homologous boosting with VLA2001 resulted in higher neutralizing antibody titers than heterologous boosting of ChAdOx1-S–primed individuals (58). Our results align with and extend these findings to the ORFV platform, reinforcing the broader principle that heterologous vaccination strategies can leverage complementary features of distinct vaccine technologies to optimize immunogenicity. Consistent with this, we found that Prime-2-CoV priming followed by VLA2001 boosting was the least immunogenic heterologous regimen, highlighting that the sequence and compatibility of platforms and antigens are critical determinants of success in heterologous vaccination approaches.

Beyond immunological efficacy, heterologous strategies offer practical advantages for public health. They increase flexibility in vaccine deployment, enable better use of existing supply chains, and may mitigate platform-specific limitations. For example, adenovirus-based vaccines have been linked to rare but serious adverse events such as vaccine-induced thrombotic thrombocytopenia, limiting their use in some populations (59).

Our results further support incorporating multiple viral antigens, beyond spike, in vaccine designs. While spike remains the primary target of neutralizing antibodies, conserved internal proteins, such as nucleocapsid, may broaden immune protection (60–62). The ability of Prime-2-CoV to strongly boost nucleocapsid-specific responses following VLA2001 priming exemplifies the benefit of dual-antigen constructs. Indeed, multiple vaccine candidates incorporating both spike and nucleocapsid have demonstrated in preclinical (16, 60, 63–65) and clinical (66–68) studies, that this strategy can substantially enhance cell-mediated immunity and promote cross-reactive T cell responses (60, 63, 64). This may provide a degree of protection less vulnerable to Spike mutations, which frequently occur in emerging variants (2, 41, 69–71). While nucleocapsid alone is reported to confer minimal (60) to no (72) protective efficacy, its inclusion alongside spike could mitigate limitations of spike-only vaccines (60, 66, 73). Although the protective contribution of nucleocapsid is not fully resolved; nevertheless, N-directed, T-cell-biased immunity has reduced viral burden or disease even in the absence of spike in a route- and context-dependent manner (69, 74). Consistent with this, several studies challenge the notion that neutralizing antibodies alone define protection and highlight potentially protective nucleocapsid-specific immune responses (60, 66, 73). Despite extensive research on SARS-CoV-2, the precise protective mechanisms associated with nucleocapsid remain incompletely understood, but proposed pathways include cytotoxic T cell responses (72, 75), NK cell-mediated antibody-dependent cellular cytotoxicity (72, 76), and Fc-mediated antibody functions (77) that enhance antigen presentation and cross-priming (75).

This study has several limitations. It is a preclinical proof−of−concept in mice without viral challenge. Vaccinations were administered intramuscularly to mirror the clinical use of both platforms; accordingly, we focused on systemic readouts and did not analyze mucosal compartments such as lung tissue, lung-draining lymph nodes, or bronchoalveolar lavage. Neutralizing antibodies were assessed using an ACE2-RBD competitive inhibition assay as opposed to pseudo- or live-virus based neutralization assays. While it does not directly measure neutralization, such assays have been used by several other groups (78–80) and have been shown to have directly comparable performance to classical neutralization assays. As they are typically bead-based and require no live cells or viruses, they offer a highly standardized method for assessing neutralizing antibody function against multiple variants simultaneously (34). Critically the assay format also means that minimal sample volumes are required making them ideally suited for use in preclinical studies where sample volumes are restricted. The study was powered for immunogenicity endpoints, follow-up ended two weeks after boost, and rare safety signals were not assessed. To enhance modest nucleocapsid-directed CD8^+^ immunity, future work should explore antigen-engineering strategies such as driving nucleocapsid expression from an early promoter. Dedicated follow-on studies are needed to evaluate standardized neutralization against current variants, to assess durability and mucosal immunity, and to establish efficacy in challenge models and ultimately clinical settings.

While our study focused on intramuscular administration to mirror clinical use, the site of acquisition for SARS-CoV-2 is the respiratory mucosa (81). Masopust and colleagues have shown that the immunization route critically shapes local immunity, with mucosal vaccination favoring airway tissue-resident memory T cells (TRM) and enhancing barrier protection against SARS-CoV-2 infection (74). Intranasal immunization with an ORFV-based rabies vaccine has previously proven effective in mice, indicating that this vector is compatible with mucosal delivery (26). Such approaches could be combined with systemic priming in a “push–pull” strategy, where intramuscular vaccination drives robust systemic immunity and subsequent mucosal boosting recruit effector and memory cells to the airway (82). This concept is particularly relevant for establishing airway TRM and secretory IgA, both of which play pivotal roles in early viral control at the site of entry (83, 84). However, substantial work remains to optimize delivery routes, dosing, and regimens for ORFV-based vaccines in this context.

Beyond SARS-CoV-2, the ORFV platform’s capacity to express multiple immunogenic antigens and induce strong Th1-biased immune responses makes it an attractive technology for vaccines against other pathogens that demand broad and durable immunity.

In summary, Prime-2-CoV is as a potent booster vaccine when combined with VLA2001 in a heterologous prime-boost regimen. This combination elicited strong spike- and nucleocapsid-specific immune responses with favorable Th1 polarization. Within the scope of this intramuscular immunogenicity screen, these data support advancing ORFV-based vectors as adaptable components of heterologous vaccination strategies beyond COVID-19. The platform warrants evaluation against other respiratory pathogens where breadth and durable T-cell immunity are priorities. Future work should establish durability and protection and, for respiratory viruses, quantify airway mucosal immunity and test targeted intranasal boosting strategies.

## Data Availability

The original contributions presented in the study are included in the article/**Supplementary Material**. Further inquiries can be directed to the corresponding author.

## Supplementary material

Supplementary Figure 1 ORFV-specific humoral responses following Prime-2-CoV vaccination in CD-1 mice. (A) ORFV-specific total IgG endpoint titers in mouse serum measured by ELISA at two weeks after the first (day 14) and two weeks after the second (day 35) immunization. Data are presented as geometric mean values ± geometric standard deviation (SD). Geometric mean titers (GMT) are noted above the columns. (B, C) Correlation of ORFV-specific total IgG endpoint titers on day 14 and day 35 versus B) spike-specific and C) nucleocapsid (N)-specific total IgG endpoint titers in mouse serum on day 35.

Supplementary Figure 2 Germinal center and antigen-specific CD4^+^ T cell responses induced by Prime-2-CoV and VLA2001 in homo- and heterologous vaccination regimens. CD-1 mice were immunized on days 0 and 21 with 10^6^ PFU of Prime-2-CoV, 1/10 of the human dose of VLA2001, or PBS. **(A)** Number of germinal center (GC) B cells and T follicular helper (Tfh) cells per spleen on day 35. **(B, C)** Number of **(B)** spike-specific and **(C)** nucleocapsid (N)-specific CD4^+^ T cells per spleen assessed on day 35 by intracellular cytokine staining (ICS) following *ex vivo* peptide restimulation. In **(A–C)** heights of bars indicate mean ± SEM (standard error of the mean).

Supplementary Figure 3 Correlation of spike- and nucleocapsid-specific immune responses following homologous and heterologous vaccination with Prime-2-CoV and VLA2001. (**A–H)** Correlation analyses of humoral and cellular immune response readouts in CD-1 mice. **(A, B)** Nucleocapsid (N)-specific total IgG endpoint titers in serum on A) day 35 and B) day 21 versus spike-specific total IgG endpoint titers on day 35. **(C)** Spike-specific total IgG endpoint titers in serum on day 21 versus N-specific total IgG endpoint titers on day 35. **(D, E)** Numbers of N-specific CD4^+^ T cells per spleen on day 35 versus **(D)** spike-specific total IgG endpoint titers in serum and **(E)** wild-type (WT) ACE2 binding inhibition in serum on day 35. **(F)** Numbers of spike-specific CD4^+^ T cells per spleen versus N-specific total IgG endpoint titers in serum on day 35. **(G, H)** Correlation between **(G)** spike-specific CD4^+^ and CD8^+^ T cell numbers per spleen and **(H)** N-specific CD4^+^ and CD8^+^ T cell numbers per spleen on day 35.
