# Supplementary material for: Heterologous prime-boost vaccination with VLA2001 and an ORFV-based vector enhances spike- and nucleocapsid-specific immunity in mice
Source: Front Immunol. 2025 Sep 18;16:1675859. doi: 10.3389/fimmu.2025.1675859 (PMC12488559; doi:10.3389/fimmu.2025.1675859)

**Supplementary Figure S1. ORFV-specific humoral responses following Prime-2-CoV vaccination in CD-1 mice. A)** ORFV-specific total IgG endpoint titers in mouse serum measured by ELISA at two weeks after the first (day 14) and two weeks after the second (day 35) immunization. Data are presented as geometric mean values ± geometric standard deviation (SD). Geometric mean titers (GMT) are noted above the columns. **B), C)** Correlation of ORFV-specific total IgG endpoint titers on day 14 and day 35 versus B) spike-specific and C) nucleocapsid (N)-specific total IgG endpoint titers in mouse serum on day 35.


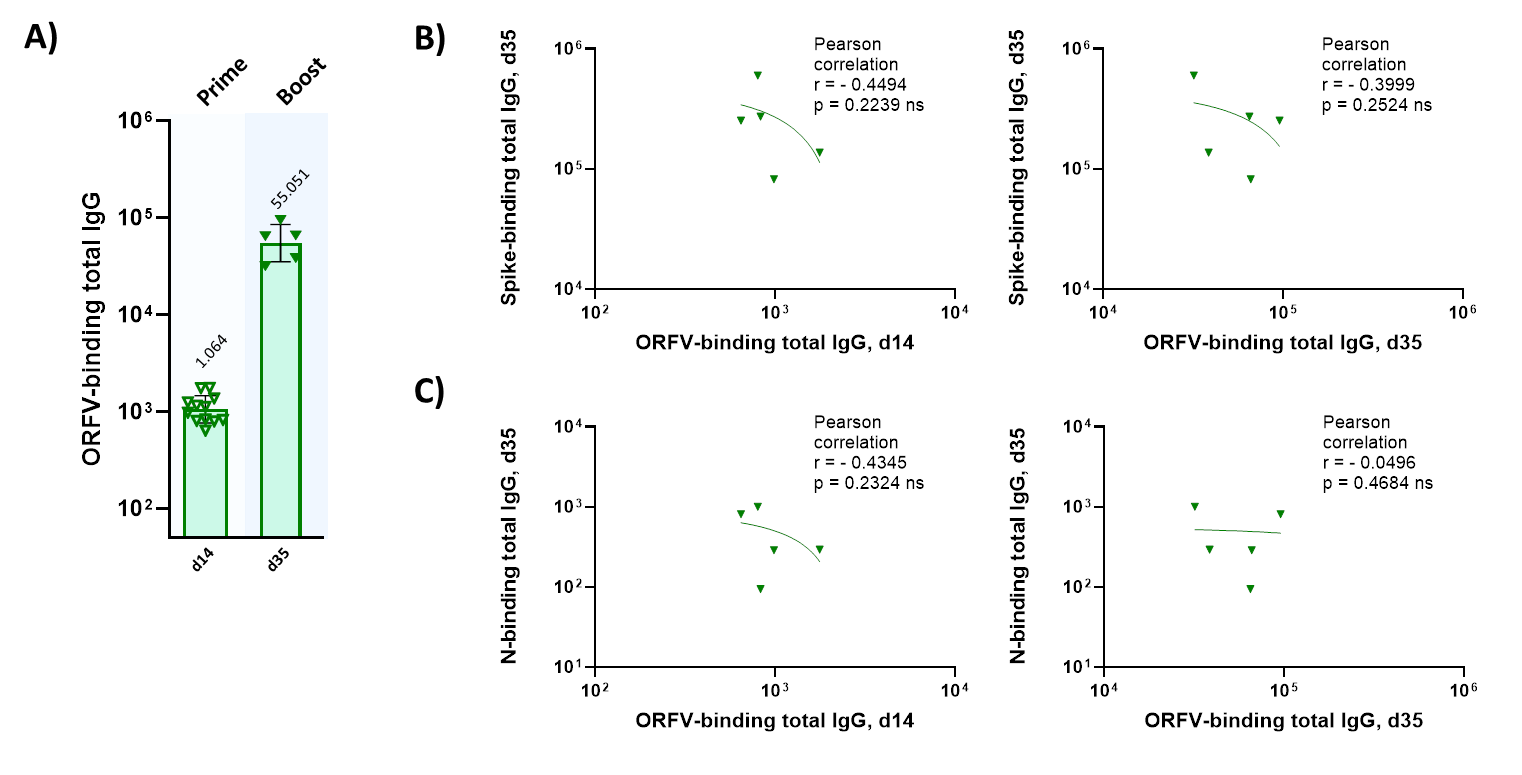


**Supplementary Figure S2. Germinal center and antigen-specific CD4⁺ T cell responses induced by Prime-2-CoV and VLA2001 in homo- and heterologous vaccination regimens.** CD-1 mice were immunized on days 0 and 21 with 10⁶ PFU of Prime-2-CoV, 1/10 of the human dose of VLA2001, or PBS. **A)** Number of germinal center (GC) B cells and T follicular helper (Tfh) cells per spleen on day 35. **B), C)** Number of B) spike-specific and C) nucleocapsid (N)-specific CD4⁺ T cells per spleen assessed on day 35 by intracellular cytokine staining (ICS) following *ex vivo* peptide restimulation. In A) – C) heights of bars indicate mean ± SEM (standard error of the mean).


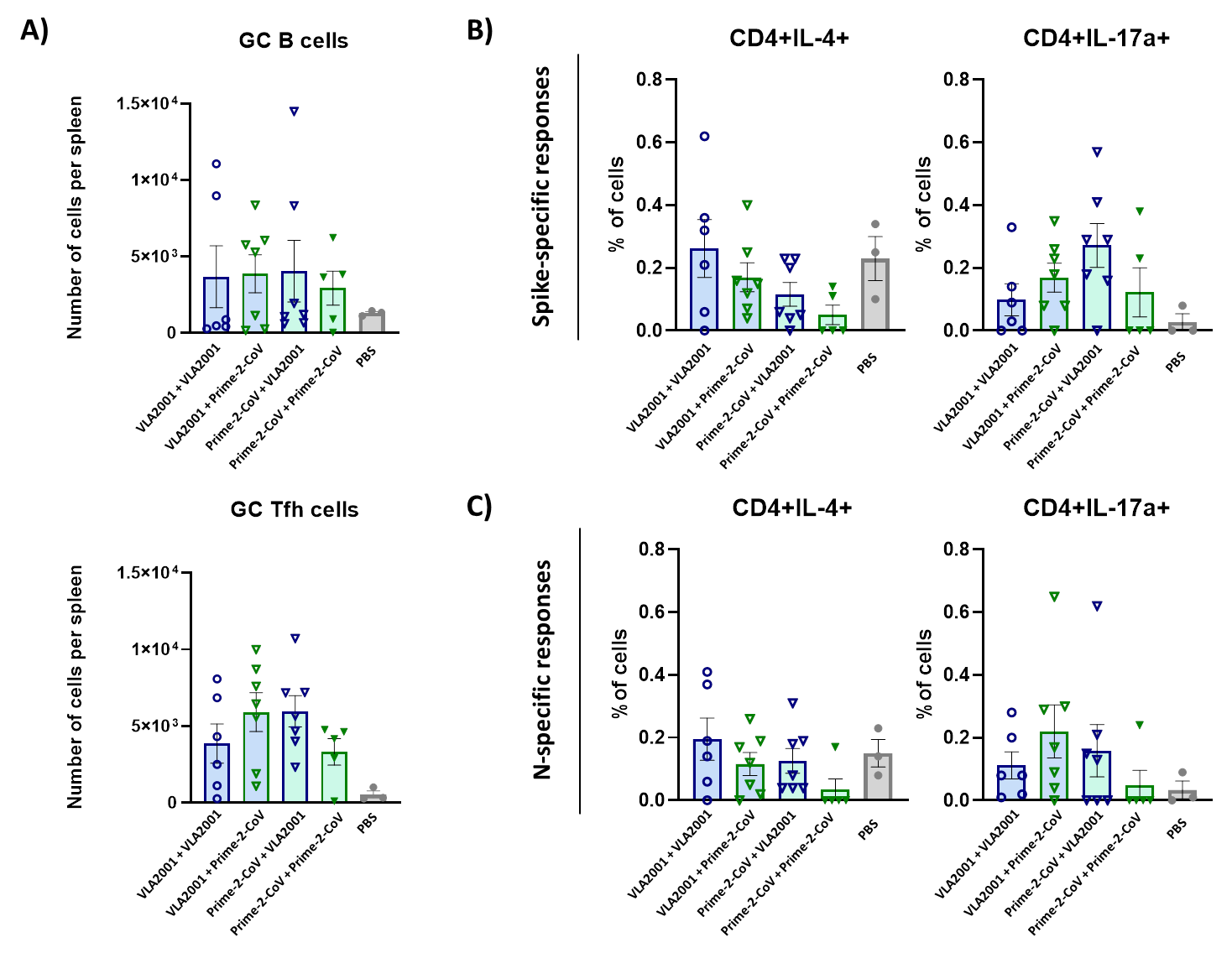


**Supplementary Figure S3. Correlation of spike- and nucleocapsid-specific immune responses following homologous and heterologous vaccination with Prime-2-CoV and VLA2001.**
**A–H)** Correlation analyses of humoral and cellular immune response readouts in CD-1 mice. **A), B)** Nucleocapsid (N)-specific total IgG endpoint titers in serum on A) day 35 and B) day 21 versus spike-specific total IgG endpoint titers on day 35. **C)** Spike-specific total IgG endpoint titers in serum on day 21 versus N-specific total IgG endpoint titers on day 35. **D), E)** Numbers of N-specific CD4⁺ T cells per spleen on day 35 versus D) spike-specific total IgG endpoint titers in serum and E) wild-type (WT) ACE2 binding inhibition in serum on day 35. **F)** Numbers of spike-specific CD4⁺ T cells per spleen versus N-specific total IgG endpoint titers in serum on day 35. **G), H)** Correlation between G) spike-specific CD4⁺ and CD8⁺ T cell numbers per spleen and H) N-specific CD4⁺ and CD8⁺ T cell numbers per spleen on day 35.


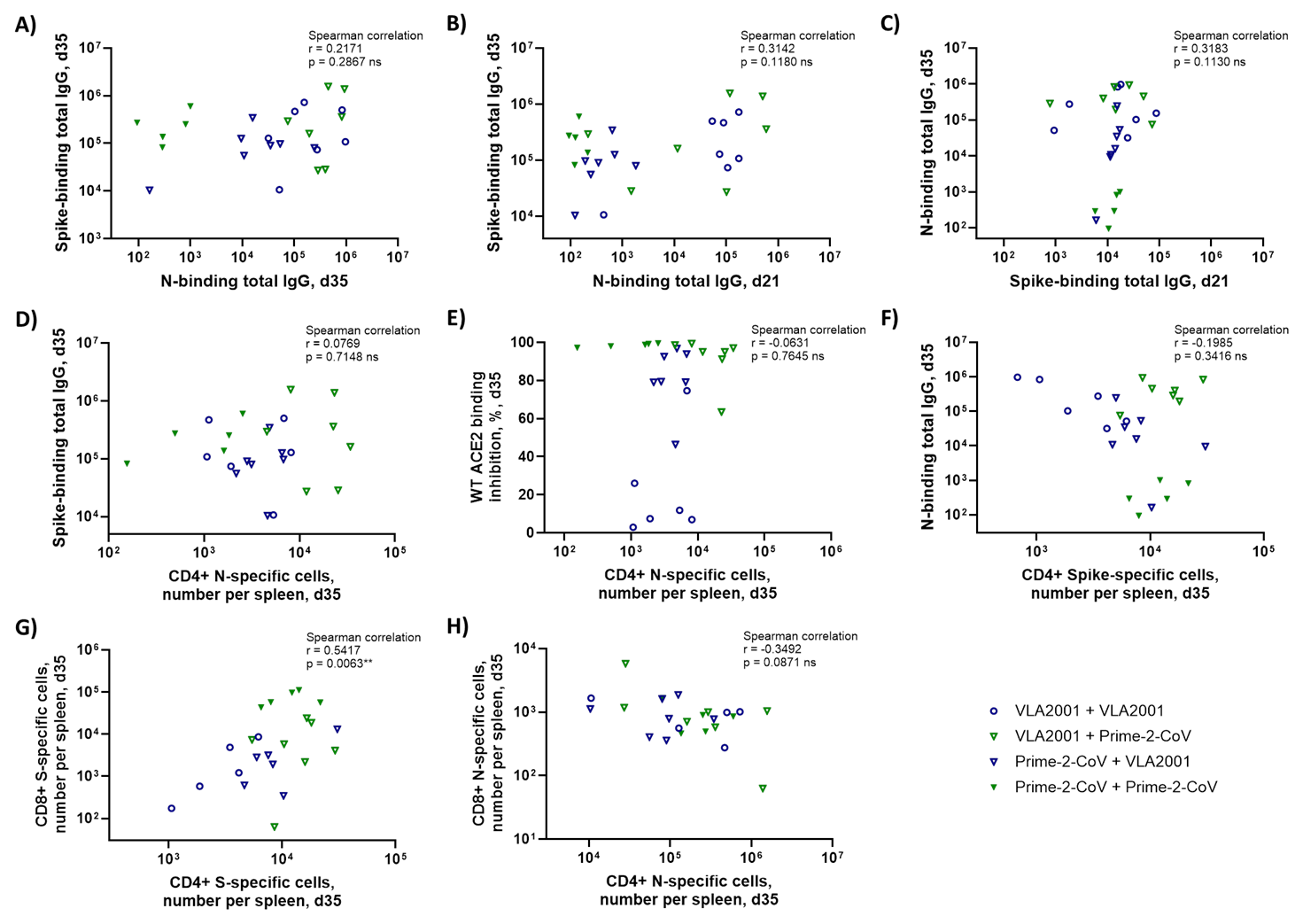

Supplement: Supplementary Figure 1 — ORFV-specific humoral responses following Prime-2-CoV vaccination in CD-1 mice. (A) ORFV-specific total IgG endpoint titers in mouse serum measured by ELISA at two weeks after the first (day 14) and two weeks after the second (day 35) immunization. Data are presented as geometric mean values ± geometric standard deviation (SD). Geometric mean titers (GMT) are noted above the columns. (B, C) Correlation of ORFV-specific total IgG endpoint titers on day 14 and day 35 versus B) spike-specific and C) nucleocapsid (N)-specific total IgG endpoint titers in mouse serum on day 35. [file DataSheet1.docx]
